# Randomised clinical trial: alosetron improves quality of life and reduces restriction of daily activities in women with severe diarrhoea-predominant IBS

**DOI:** 10.1111/j.1365-2036.2012.05208.x

**Published:** 2012-07-10

**Authors:** F Cremonini, J P Nicandro, V Atkinson, R Shringarpure, E Chuang, A Lembo

**Affiliations:** * Division of Gastroenterology, Beth Israel Deaconess Medical Center, Harvard Medical SchoolBoston, MA, USA; † Southern Nevada VA Healthcare SystemLas Vegas, NV, USA; ‡ Prometheus Laboratories Inc.San Diego, CA, USA

## Abstract

**Background:**

Patients with irritable bowel syndrome with diarrhoea (IBS-D) experience restriction in daily activities and decreased health-related quality of life (QOL).

**Aim:**

To investigate effects of alosetron on patient-reported health-related QOL, satisfaction and productivity in women with severe IBS-D.

**Methods:**

A total of 705 women (severe IBS-D, Rome II criteria) randomised to alosetron 0.5 mg QD, 1 mg QD, 1 mg BID, or placebo for 12 weeks were studied. IBSQOL, treatment satisfaction, daily activities, and lost workplace productivity (LWP) were evaluated at randomisation and Week 12.

**Results:**

One or more doses of alosetron significantly improved all IBSQOL domains except for sexual function from baseline vs. placebo. The magnitude of IBSQOL changes was consistent with a clinically meaningful effect. Alosetron 0.5 mg QD and 1 mg BID significantly reduced IBS interference with social/leisure activities and LWP from baseline vs. placebo [social/leisure (mean ±S.E.) days lost: −6.7 ± 0.8, −7.0 ± 0.9, *P* < 0.01; LWP (mean ± S.E.) h lost: −11.0 ± 3.3, −21.1 ± 4.1, *P* < 0.05 respectively]. Significantly more patients treated with alosetron reported satisfaction vs. placebo. Improvements in IBSQOL, LWP, and treatment satisfaction significantly correlated with global improvement of IBS symptoms. The incidence of adverse events with alosetron was low with constipation being the most commonly reported event. A single case of ischaemic colitis occurred, in a patient receiving alosetron 0.5 mg QD.

**Conclusions:**

In women with severe IBS-D, alosetron treatment, including 0.5 mg QD, resulted in statistically significant and clinically relevant improvements in health-related QOL, restriction of daily activities and treatment satisfaction over placebo. IBS symptom improvement corresponded with positive changes in IBSQOL, LWP and treatment satisfaction.

## Introduction

The impact of the irritable bowel syndrome (IBS) reaches beyond abdominal pain and altered bowel habits. Patients suffering with IBS, compared to individuals without IBS, generally experience differing levels of restriction in daily activities (e.g. diminished workplace productivity and reduced physical/social functioning), worse psychological well-being and lower quality of life. The estimated total direct and indirect healthcare costs associated with IBS are sixfold higher compared to healthy individuals,^1^, ^2^ and IBS patients are more likely to undergo unnecessary abdominal surgeries than individuals without IBS.^3^, ^4^ The burden of IBS on psychosocial, economic, quality of life and daily function aspects is even greater in patients with severe and refractory symptoms.^5^ The estimated proportion of IBS patients considered to have severe symptoms ranges from 20% to 55%.^6^

Although treatment includes dietary and lifestyle modifications, patients with severe IBS with diarrhoea (IBS-D) frequently require additional pharmacological therapy. The choice of treatment is influenced by predominant bowel habit, symptom severity and the impact of symptoms on quality of life. Disappointingly, the treatment options for patients with severe IBS-D remain limited.

Alosetron is a selective 5-HT_3_ receptor antagonist that has been shown to significantly improve abnormal bowel function and to relieve pain and discomfort in IBS-D.^7^–^18^ Alosetron, at the 1 mg twice daily (BID) dose, improved quality of life and workplace productivity in previous randomised, controlled trials.^19^–^21^ These effects were associated with significant overall treatment satisfaction.^19^ The current recommended starting dose of alosetron is 0.5 mg BID, which can be decreased to 0.5 mg QD if significant constipation occurs. Therefore, the effect of the 0.5 mg QD dose of alosetron on patient-reported health outcome measures warrants clinical evaluation.

Ischaemic colitis and complications of constipation are known serious adverse events associated with alosetron; concern over these adverse events has led to the use of the drug under a Risk Evaluation and Mitigation Strategy (REMS) programme. Constipation is the most common adverse event observed with alosetron treatment, which occurs more frequently with increasing dosages. The incidence of ischaemic colitis and complications of constipation do not appear to be dose-related.^22^

The aim of the present study was to evaluate the effect of alosetron on IBS-related quality of life, restriction of daily activities (workplace productivity, social/leisure activity, household activity), and treatment satisfaction in women with severe IBS-D who had inadequate responses to other IBS-D therapies in a randomised, double-blind, placebo-controlled study that included lower doses of alosetron than previously reported.

## Materials and Methods

Patients were enrolled from June 2003 to June 2005 at multiple study sites in the United States. Institutional Review Boards at each individual site approved the protocol. Patients provided written informed consent upon study entry. The efficacy data of alosetron on IBS symptom relief from this study have been previously reported.^15^ Details of the clinical trial are publicly accessible at clinicaltrials.gov, registration number: NCT00067561.

### Participants

Women aged 18 years or older, diagnosed with severe IBS-D [at least 6 months of IBS-D symptoms as defined by the Rome II criteria^23^] who had failed conventional therapy were eligible for the study. In addition, patients were required to report at least one of the following symptoms to be eligible for the study:

- Severe IBS-D defined as either diarrhoea occurring ≥50% of the time with an average stool consistency score ≥3.0, or an average stool consistency score ≥3.5. The assessment of stool consistency was based on a 5-point scale (1 = very hard, 2 = hard, 3 = formed, 4 = loose, 5 = watery).
- In addition to meeting the diarrhoea and stool consistency criteria, patients also needed to report at least one of the following symptoms during the 2-week screening period to be eligible for the study:
  - Frequent and severe abdominal pain/discomfort (average pain score ≥2.0; 5-point scale: 0 = none, 1 = mild, 2 = moderate, 3 = intense, 4 = severe); or
  - Frequent bowel urgency or faecal incontinence (urgency ≥ 50% of days); or
  - Restriction of daily activities because of IBS (≥25% of the days).

Patients were required to discontinue use of IBS medication for 7 days prior to screening with the exception of short-acting antidiarrheals (e.g. loperamide or diphenoxylate), which could be taken ≤24 h prior to screening, if necessary. Antidepressants, with the exception of mirtazapine were permitted.

### Exclusion criteria

Exclusion criteria were the presence of a biochemical or structural abnormality of the digestive tract (including ischaemic colitis, adhesions and impaired intestinal circulation); concomitant unstable medical condition; or history or evidence of bloody diarrhoea or abdominal pain with rectal bleeding, chronic or severe constipation, or complications from constipation. Patients who were currently constipated or had no stool for three consecutive days during the 2-week screening period were also excluded.

### Randomisation

Patients satisfying all inclusion and exclusion criteria were randomly assigned (1:1:1:1) to placebo BID, alosetron 0.5 mg QD (plus placebo QD), alosetron 1 mg QD (plus placebo QD), or alosetron 1 mg BID for 12 weeks. Patients and all study site personnel were blinded to treatment assignment. Patients were assigned to study treatment in accordance with a central, blocked randomisation schedule. Study drug compliance was assessed for each patient using pill counts and recorded via the telephone diary system.

All analyses were conducted using the intent-to-treat (ITT) population which consisted of all subjects randomised to treatment. The last observation carried forward (LOCF) imputation method was used for missing values. In the LOCF approach, missing responses were replaced with the value of the last previous non-missing response.

### Assessments

Quality of life data were collected via a disease-specific questionnaire (IBSQOL) at Randomisation and Week 12, using a recall period of the preceding 4 weeks. The IBSQOL is designed to assess the impact of IBS on nine dimensions of health status, including emotional functioning, mental health, sleep behaviour, energy, physical functioning, diet, social role, role physical and sexual relations.^24^ The IBSQOL has demonstrated validity and reliability in prior prospective studies in IBS. Questionnaires to assess workplace productivity, restriction of daily activities (i.e. household and social/leisure activities) and treatment satisfaction were also administered at randomisation and Week 12.

Lost workplace productivity (LWP), including time lost from paid job(s) and reduced effectiveness at paid job(s) due to IBS symptoms were assessed at baseline and Week 12 by asking patients to recall the impact of IBS symptoms during the preceding 4 weeks. LWP was assessed only in subjects scheduled to work during the study (approximately two thirds of the ITT population). LWP was calculated as follows:


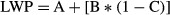


where A = Hours missed from paid job(s) because of IBS symptoms. B = Hours working at paid job(s) despite experiencing interference of IBS symptoms. C = Per cent effectiveness while working with symptoms at paid job(s).

Restriction of daily activities was evaluated through measurement of interference of IBS symptoms with social/leisure and household activities, which were also assessed at randomisation and Week 12 by asking the patient questions recalling the impact of IBS symptoms during the preceding 4 weeks.

### Treatment satisfaction

Patient treatment satisfaction was assessed at randomisation and Week 12 by the following question: ‘During the past 4 weeks, how satisfied were you with the medication(s) you have used to treat your IBS symptoms?’ and recorded a satisfaction score for their IBS medication using a 7-point scale ranging from 1 = very satisfied to 7 = very unsatisfied.

### Correlation with efficacy on IBS symptom relief

Efficacy results of alosetron on the IBS global improvement scale (GIS) in this study have been previously published.^15^ We examined correlations between the changes from baseline in quality of life domains, LWP, treatment satisfaction and the GIS responses obtained.

### Statistical analysis

All data analyses were performed using sas (V8.2; copyright 1999–2001 SAS Institute, Cary, NC, USA). Changes from baseline in scale-specific scores were computed for all nine quality of life domains: energy, social functioning, mental health, physical role, physical functioning, emotional, food/diet, sleep and sexual relations. For quality of life data, change from baseline to Week 12 in transformed scale-specific scores were analysed using analysis of covariance^25^ with effects for treatment and baseline transformed scale score as the covariate. Pair-wise comparisons of each alosetron dose group versus placebo were adjusted for the nine IBSQOL scales using the general multiple Simes procedure described by Hommel.^26^

Change from baseline in LWP, and in the number of days IBS symptoms interfered with social/leisure and household activities were summarised and compared between treatment groups using analysis of covariance^25^ following rank transformation of the data with the baseline value as a covariate.

Treatment satisfaction scores between groups were compared at Week 12 (ITT-LOCF) using the Wilcoxon rank sum test.^27^ Also, for each treatment group, medication satisfaction with assigned treatment at Week 12 was compared to the satisfaction of the previously used regimen at baseline using the sign-rank test.^28^

Responder status and ratings for GIS at Week 12 were correlated with IBSQOL, workplace productivity and treatment satisfaction parameters using Spearman's rank correlation coefficient.^28^ Summary statistics (mean, s.d.) of the changes in individual and aggregate quality of life scales that were associated with positive changes in the GIS were also obtained.

## Results

### Demographics

**Figure 1:**
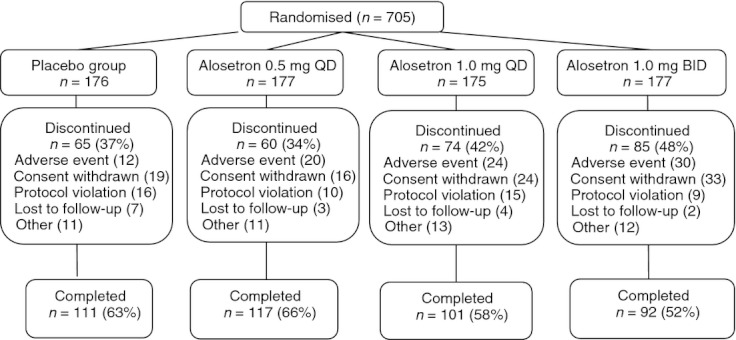
Patient disposition. QD, once daily; BID, twice daily.

Figure 1 describes the progression of patients in the study. Full details of patients screened have been previously published.^15^ Of the 705 women with severe IBS-D who were randomised, at least 52% of patients in each treatment group completed the study. The primary reasons for premature discontinuation were adverse events (86 patients, 12%) and withdrawal of consent (92 patients, 13%). Compliance with study treatment evaluated from the telephone data entry system was similar between groups, with 87–93% of patients reporting >80% compliance.

There were no significant differences in demographic features, including IBS symptom severity between the treatment arms at baseline (Table 1). All patients reported restriction of daily activities from IBS symptoms on ≥25% of days with actual restrictions on 68–70% of days.

**Table 1:** Summary of demographic and baseline characteristics of study patients

	Placebo *n* = 176*	Alosetron 0.5 mg QD *n* = 177*	Alosetron 1 mg QD *n* = 175*	Alosetron 1 mg BID *n* = 177*
Age (y) (Mean ± s.d.)	46.6 ± 14.5	45.9 ± 13.1	46.6 ± 12.3	47.0 ± 13.9
Ethnic origin [*n* (%)]				
Caucasian	154 (88)	163 (92)	158 (90)	145 (87)
Black	10 (6)	8 (5)	5 (3)	10 (6)
Hispanic	10 (6)	6 (3)	10 (6)	12 (7)
Others	2 (1)	(0)	2 (1)	(0)
Quality of Life Scores (Mean ± S.E.)				
Emotional	37.5 (1.8)	34.6 (1.7)	36.4 (1.9)	36.4 (1.7)
Mental health	57.8 (1.8)	58.3 (1.8)	58.9 (2.0)	58.7 (1.9)
Sleep	59.3 (1.8)	57.5 (1.8)	58.6 (1.8)	59.1 (1.8)
Energy	40.8 (2.0)	37.5 (2.0)	38.6 (2.0)	38.7 (1.8)
Physical functioning	56.3 (1.8)	55.9 (1.8)	57.6 (2.1)	55.5 (2.0)
Food/diet	48.7 (1.7)	44.9 (1.7)	46.0 (1.9)	46.9 (1.7)
Social functioning	32.0 (1.7)	31.5 (1.7)	32.1 (1.8)	32.3 (1.8)
Physical role	29.4 (1.8)	32.0 (1.8)	31.3 (1.8)	30.2 (1.8)
Sexual relations	53.9 (2.8)	51.8 (2.4)	52.7 (2.6)	55.9 (2.5)
Lost workplace productivity (h) (Mean ± S.E.)	31.2 (3.0)	33.5 (3.0)	32.2 (3.2)	34.6 (4.2)

BID, twice daily; ITT, intent-to-treat; QD, once daily.

* ITT population (*N* = 705).

### Health-related quality of life changes

Statistically significant improvements were observed for at least one alosetron dose in all IBSQOL domains relative to placebo, with the exception of sexual relations (Table 2).

**Table 2:** IBSQOL scores [mean (S.E.)] for the alosetron treatment groups and placebo at baseline and Week 12

Domain	Placebo *n* = 176*	Alosetron 0.5 mg QD *n* = 177*	Alosetron 1 mg QD *n* = 175*	Alosetron 1 mg BID *n* = 177*
Emotional				
*n*†	170	170	169	166
Baseline	37.5 (1.8)	34.6 (1.7)	36.4 (1.9)	36.4 (1.7)
Change from baseline	16.3 (1.9)	26.5 (1.9)	21.4 (1.9)	23.4 (2.0)
Treatment difference	–	10.1 (2.7)	5.1 (2.7)	7.1 (2.8)
*P*-value‡	–	0.001	0.066	0.021
Mental health				
*n*†	170	171	169	166
Baseline	57.8 (1.8)	58.3 (1.8)	58.9 (2.0)	58.7 (1.9)
Change from baseline	10.2 (1.6)	19.7 (1.6)	14.9 (1.6)	17.3 (1.6)
Treatment difference	–	9.5 (2.2)	4.7 (2.2)	7.0 (2.2)
*P*-value‡	–	<0.001	0.036	0.003
Sleep				
*n*†	176	177	174	171
Baseline	59.3 (1.8)	57.5 (1.8)	58.6 (1.8)	59.1 (1.8)
Change from baseline	13.4 (1.5)	18.4 (1.5)	16.6 (1.5)	18.7 (1.5)
Treatment difference	–	5.0 (2.1)	3.2 (2.1)	5.3 (2.2)
*P*-value‡	–	0.038	0.141	0.029
Energy				
*n*†	176	177	174	171
Baseline	40.8 (2.08)	37.5 (2.0)	38.6 (2.0)	38.7 (1.8)
Change from baseline	17.4 (2.0)	31.6 (2.0)	25.8 (2.0)	28.1 (2.1)
Treatment difference	–	14.2 (2.9)	8.4 (2.9)	10.7 (2.9)
*P*-value‡	–	<0.001	0.004	<0.001
Physical functioning				
*n*†	175	171	170	165
Baseline	56.3 (1.8)	55.9 (1.8)	57.6 (2.1)	55.5 (2.0)
Change from baseline	15.7 (1.6)	21.9 (1.7)	19.6 (1.7)	20.1 (1.7)
Treatment difference	–	6.2 (2.3)	3.9 (2.3)	4.4 (2.4)
*P*-value‡	–	0.024	0.094	0.094
Food/diet				
*n*†	176	177	174	170
Baseline	48.7 (1.7)	44.9 (1.7)	46.0 (1.9)	46.9 (1.7)
Change from baseline	13.7 (1.7)	22.8 (1.7)	19.1 (1.7)	19.8 (1.7)
Treatment difference	–	9.1 (2.4)	5.4 (2.4)	6.1 (2.4)
*P*-value‡	–	<0.001	0.023	0.02
Social functioning				
*n*†	175	177	174	171
Baseline	32.0 (1.7)	31.5 (1.7)	32.1 (1.8)	32.3 (1.8)
Change from baseline	16.9 (1.9)	27.0 (1.9)	22.9 (2.0)	26.5 (2.0)
Treatment difference	–	10.0 (2.7)	6.0 (2.8)	9.6 (2.8)
*P*-value‡	–	0.001	0.031	0.001
Physical role				
*n*†	175	177	173	171
Baseline	29.4 (1.8)	32.0 (1.8)	31.3 (1.8)	30.2 (1.8)
Change from baseline	17.3 (2.1)	28.5 (2.1)	23.5 (2.1)	24.7 (2.1)
Treatment difference	–	11.2 (2.9)	6.2 (3.0)	7.5 (3.0)
*P*-value‡	–	<0.001	0.036	0.024
Sexual relations				
*n*†	89	104	97	103
Baseline	53.9 (2.8)	51.8 (2.4)	52.7 (2.6)	55.9 (2.5)
Change from baseline	11.4 (2.4)	16.6 (2.2)	18.0 (2.3)	18.7 (2.2)
Treatment difference	–	5.3 (3.2)	6.7 (3.3)	7.3 (3.2)
*P*-value‡	–	0.103	0.084	0.063

BID, twice daily; ITT, intent-to-treat; LOCF, last observation carried forward; QD, once daily.

* ITT population (*n* = 705); scores presented for baseline, change from baseline, and treatment difference are mean (S.E.).

† LOCF imputed from the ITT population.

‡ Change from baseline to Week 12 in transformed scale-specific scores were analysed using analysis of covariance with effects for treatment and baseline transformed scale score as the covariate. *P*-values were adjusted for multiple comparisons using the general multiple Simes procedure described by Hommel.

### Correlation of health-related quality of life changes with IBS symptom improvement

Correlations between Week 12 global improvement of IBS symptoms and changes from baseline in the nine IBSQOL domains are reported in Table 3. For all nine IBSQOL scales, improvements in change from baseline scores were significantly correlated with Week 12 global improvement of IBS symptoms.

**Table 3:** Correlations between improvements in changes from baseline in quality of life domains, workplace productivity and treatment satisfaction vs. Week 12 global improvement of IBS symptoms. Results reported were all statistically significant and moderately correlated

Outcome measure	Spearman coefficient*	*P*-value
Quality of life domain		
Emotional	0.53	<0.0001
Mental health	0.44	<0.0001
Sleep	0.38	<0.0001
Energy	0.51	<0.0001
Physical functioning	0.40	<0.0001
Food/diet	0.51	<0.0001
Social functioning	0.57	<0.0001
Physical role	0.53	<0.0001
Sexual relations	0.45	<0.0001
Lost workplace productivity	−0.34	<0.0001
Treatment satisfaction	−0.56	<0.0001

GIS, global improvement scale; IBS, irritable bowel syndrome; IBSQOL, irritable bowel syndrome quality of life.

* Responder status and ratings for GIS at Week 12 were correlated with IBSQOL, workplace productivity and treatment satisfaction parameters using Spearman's rank correlation coefficient.

### Productivity

**Figure 2:**
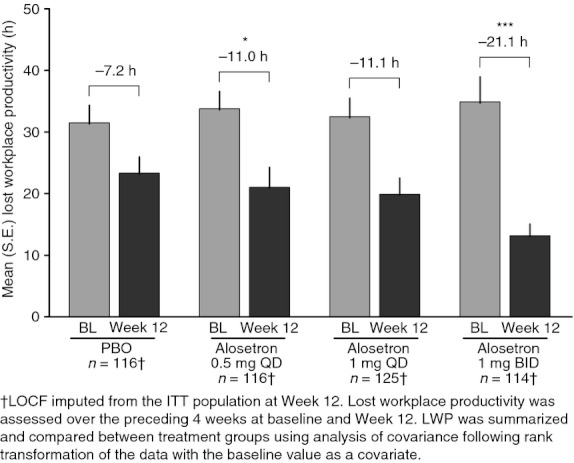
Mean number of hours of lost workplace productivity at baseline and Week 12 for the placebo (PBO) and alosetron treatment groups. BL, baseline; QD, once daily; BID, twice daily. *P < 0.05, ***P < 0.001, significantly different from placebo.

Statistically significant reductions in LWP were observed in the 0.5 mg QD (decrease of 11.0 h; *P* < 0.05) and the 1 mg BID (decrease of 21.1 h; *P* < 0.001) groups compared with placebo (decrease of 7.2 h) (Figure 2). The change from baseline in LWP was negatively correlated with the Week 12 global improvement of IBS symptoms (Table 3), suggesting that reductions in LWP were associated with improvements in global IBS symptoms.

**Figure 3:**
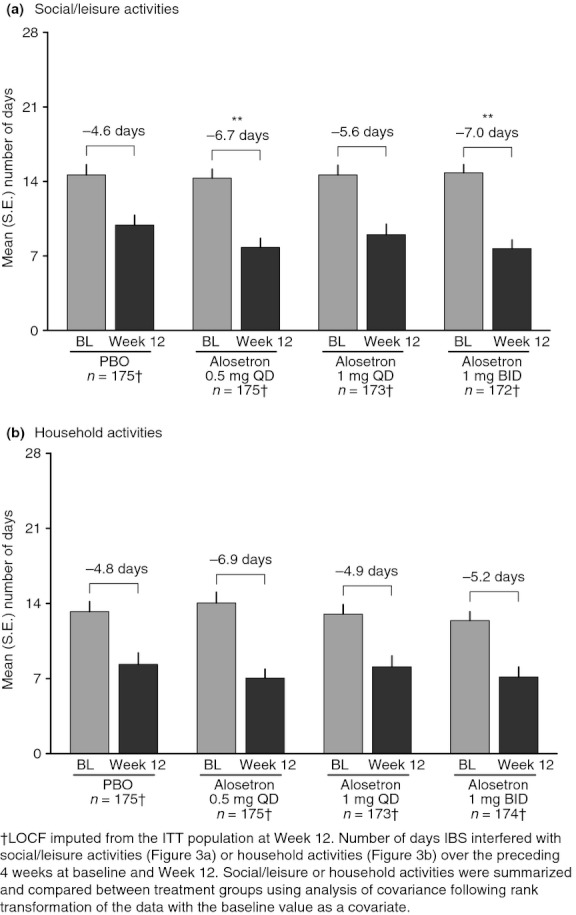
Mean number of days IBS symptoms interfered with (a) social/leisure and (b) household activities at baseline and Week 12 for the placebo (PBO) and alosetron treatment groups. BL, baseline; QD, once daily; BID, twice daily. **P < 0.01, significantly different from placebo.

The number of days IBS symptoms caused restriction of daily activities (interfered with social/leisure activities) was significantly reduced in the 0.5 mg QD and 1.0 mg BID alosetron groups compared to placebo (*P* < 0.01) (Figure 3a). Although the number of days IBS symptoms interfered with household activities was reduced, the treatment differences did not reach statistical significance compared with placebo (Figure 3b).

### Treatment satisfaction

**Figure 4:**
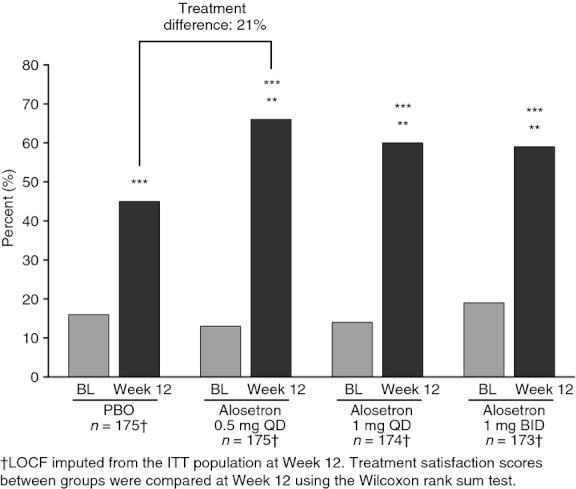
Proportion of patients satisfied with their IBS treatment at baseline and Week 12 for the placebo (PBO) and alosetron treatment groups. BL, baseline; QD, once daily; BID, twice daily. **P < 0.01 significantly different from placebo, ***P < 0.0001 significantly different from baseline.

Significant improvements in treatment satisfaction from baseline to Week 12 were observed in all treatment groups (*P* < 0.0001). At Week 12, significantly higher proportions of subjects in each alosetron dose group (*P* < 0.01) reported better satisfaction with their IBS medication compared with the placebo group (Figure 4). The number of subjects expressing treatment satisfaction with placebo increased from baseline by 29% to 45% after 12 weeks, representing a treatment difference of 21% for alosetron 0.5 mg QD vs. placebo at Week 12 (Figure 4). Greater satisfaction with IBS treatment at Week 12 was correlated with the Week 12 global improvement of IBS symptoms (Table 3).

### Safety

Safety results for this study have been previously reported.^15^ In summary, incidences of adverse events were similar across all treatment groups, with constipation being the most commonly observed adverse event. Patients experiencing adverse events were included in the analyses of patient-reported outcomes in this study. The incidence of constipation was dose-related in the alosetron groups (9%, 16%, and 19%, for the 0.5 mg QD, 1 mg QD and 1 mg BID groups respectively). Three serious adverse events were reported that were considered by investigators to be related to alosetron [two patients on 0.5 mg QD (bowel obstruction, ischaemic colitis]; one patient on 1 mg BID (faecal impaction)]. All three adverse events resolved shortly after discontinuation of treatment.

## Discussion

This randomised, placebo-controlled study demonstrated that alosetron administration, including 0.5 mg QD, was associated with improved measures of IBS-related quality of life and treatment satisfaction in women with severe IBS-D. Moreover, the 0.5 mg QD dose of alosetron was associated with improvements in lost workplace productivity, and resulted in fewer days of restriction of daily activities caused by IBS symptoms. The effects of alosetron on quality of life, workplace productivity and restriction of daily activities were moderately correlated with drug efficacy, as reflected by improvement of global IBS symptoms. Since there are limited clinical trial data on the important association between symptom improvement and health outcomes in IBS patients, it is significant when therapeutic responses to IBS treatments are reported to correspond with positive changes in health-related quality of life measures.^29^

The clinical importance of the adverse impact of IBS on patients’ ability to function normally, and on their sense of well-being, is increasingly recognised by healthcare professionals. The negative consequences of IBS on quality of life, productivity and daily activities worsen with disease severity, intensity, duration and frequency of doctor visits^30^ as patients run out of effective treatment options for their severe abdominal and bowel symptoms.

Alosetron is the only prescription medication approved by the FDA for treatment of severe IBS-D in women who have failed conventional treatment. The dosing regimen for alosetron can be adjusted depending on patient response from an initial starting dose of 0.5 mg BID to a maximum of 1 mg BID.

In the present study, the improvement in quality of life, compared to placebo, was present in eight of nine domains and generally appeared more marked at the lowest dose of alosetron. Sexual dysfunction did not improve for reasons that are not clear; however, it is of note that the sample population was lower than in the other eight domains, possibly because patients were uncomfortable answering questions regarding sexual activity. However, improvement in sexual dysfunction trended towards statistical significance with alosetron 1 mg QD (*P* = 0.084) and 1 mg BID (*P* = 0.063) doses (Table 2). The observed differences between active treatment groups and placebo in scores for individual quality of life domains ranged between 11% and 75% approximately (percentage treatment difference: active group change from baseline – placebo change from baseline/change from baseline for active group*100). Changes from baseline scores of 18 points in a similar quality of life instrument were previously found to be statistically significant and clinically relevant in a controlled trial of cognitive-behavioural treatment in IBS,^31^ and a minimum change of 14 in quality of life scores was found to be clinically meaningful in association with pharmacological or psychological treatments for IBS patients.^32^ The magnitude of improvements observed with alosetron in this study was larger than that determined to be relevant previously (however, the improvement of IBSQOL in the placebo group should also be recognised). This suggests that the effects of alosetron on quality of life reported in this study are robust and clinically meaningful.

A positive effect of alosetron on overall productivity was also observed, with the highest dose of alosetron being associated with the largest number of work hours gained and the least interference with normal daily activities. Improvements observed with the alosetron 0.5 mg QD dose resulted in approximately 50% more hours of work gained (approximately 4 h gained), and 45% fewer days of IBS symptom interference compared with placebo. This compared to an earlier prospective, natural history study, in which IBS patients lost approximately 35% productivity per 40-h workweek,^33^ and of a survey of 1776 employees in a US banking institution, where IBS patients lost an average of 15% of work hours.^34^ The effects of alosetron appear relevant, even at 0.5 mg QD, which generally produced comparable effects to the 1 mg BID dose in this study.

In this study the intermediate (1 mg QD) dose of alosetron did not have significant effects on physical functioning, emotional, sleep, or sexual relations quality of life domains, workplace productivity, or restriction of daily activities. This may indicate that side effects at the 1 mg QD dose may detract from perceived treatment benefit, whereas at the largest 1 mg BID dose the magnitude of physiological improvement on pain and bowel function may overshadow the impact of side effects, resulting in greater overall improvement in quality of life, workplace productivity, restriction of daily activities and treatment satisfaction. However, these conclusions are speculative as the determinants of quality of life, productivity, lifestyle activities and medication satisfaction remain innumerable.

The effects of alosetron 1 mg BID on quality of life and workplace productivity reported in this study are consistent with previous data.^19^–^21^ However, the magnitude of improvements elicited by alosetron was generally greater in this study, possibly due to the larger treatment response that may be expected in patients with severe IBS-D. However, an alternative explanation may be regression to the mean, as symptom and IBSQOL scores were greater at baseline.

In this study, summary values of changes in the individual domains of IBSQOL, lost workplace productivity, daily activities and treatment satisfaction are reported; these changes correspond to clinically relevant improvements of IBS symptoms. The greatest positive changes in moderate or substantial improvement of global symptoms after 12 weeks of treatment occurred in the energy domain of the IBSQOL, however, a positive change was reported in all evaluated components. The summary data reported in this study may provide the basis for planning and interpretation of future studies assessing the effect of therapeutics on measures of quality of life, workplace productivity, daily activities and treatment satisfaction in IBS patients.

There are potential limitations to the interpretation of the data presented in this study. First, the trial population is entirely women, a gender imbalance that is characteristic of IBS and its referral pattern; moreover, alosetron is currently labelled exclusively for female use. Clinical studies in men suggest similar effects on adequate relief of pain and discomfort,^17^ however, there are currently no studies formally assessing the effect of alosetron on quality of life, productivity and lifestyle in male patient populations. Second, global improvement endpoints used in alosetron studies have been criticised as not being fully representative of the complexity of IBS. Indeed, more recent FDA guidance on acceptable IBS clinical trial endpoints recommends use of a primary endpoint that specifically measures the effect of treatment on two major IBS symptoms (i.e. abdominal pain intensity and stool consistency for IBS-D).^35^, ^36^ Nonetheless, global endpoints continue to show validity in large studies with IBS-D patients,^37^ and Krause and colleagues previously reported the correlation of global improvement in IBS symptoms with relief of pain, discomfort, stool form and other gastrointestinal symptoms,2007 supporting the adequacy and usefulness of the GIS primary endpoint. Third, the authors observed adverse events even at lower doses of alosetron. Ischaemic colitis and complications of constipation are the most severe adverse events reported with alosetron use.^38^, ^39^ Ischaemic colitis appears to have a higher background incidence in patients with a diagnosis of IBS than in the general population, irrespective of IBS medical treatment.^40^ In this study, adverse events were self-limiting and resolved without long-term sequelae. A recent review^22^ summarised the clinical outcomes of ischaemic colitis and complications of constipation in patients with severe IBS-D under alosetron treatment. The cases were reviewed in the framework of a risk management plan, established since the reintroduction of alosetron to the US market in 2002. All cases reported in patients with severe IBS-D resolved or improved upon withdrawal of alosetron without serious sequelae.^22^

Not all adverse events of alosetron are correlated to drug dose. Although constipation appears to be dose-dependent, ischaemic colitis and complications of constipation do not seem to be. In this large study, a single case of reversible ischaemic colitis was observed at the lower 0.5 mg QD dose of alosetron.

In this study, we observed a substantial number of dropouts across all treatment groups. Although this could suggest a relationship with poor treatment tolerability in the highest dose group, a 37% dropout rate was observed in the placebo group, suggesting that a disorder such as severe IBS-D itself would, at least in part, explain such high rates of discontinuation.

The potential bias of the LOCF imputation method was assessed by comparison to the analysis of GIS responders using observed data with no imputation, and analysis of the data imputed as missing equals non-responder. These comparisons were similar to the LOCF imputation method, except for the Week 12 missing equals non-responder comparison between alosetron 1 mg BID and placebo (data not shown). These results indicate minimal bias in the LOCF imputation method towards the treatment difference in the proportion of GIS responders at Weeks 4 and 8; however, at Week 12 some impact was apparent due to the greater amount of missing data for the alosetron 1 mg BID group. Importantly, it should be noted that changes in quality of life domains, workplace productivity and treatment satisfaction at Week 12 were moderately correlated to GIS based on the ITT-LOCF analyses, thereby suggesting minimal bias across these study end points.

The evidence that ischaemic colitis is reversible in the setting of alosetron administration is reassuring. Moreover, the data presented in this study are a reminder of the likely idiopathic mechanisms underlying the development of ischaemic colitis. This point is further supported by the rare and stable incidence rates of ischaemic colitis associated with alosetron treatment since the initial marketing period to post reintroduction.^22^ Nonetheless, the evidence of the positive effect of a lower dose of alosetron on bowel symptom relief and meaningful effects on global IBS symptoms provide clinicians with greater flexibility in alosetron dosing.

In summary, this study demonstrates that treatment with alosetron across multiple doses, including once daily, leads to significant improvements in quality of life, work productivity, social/leisure activities and treatment satisfaction in women with severe IBS-D. Moreover, these changes were moderately and significantly correlated with improvements in global IBS symptoms.
